# Real-time magnetic resonance cine imaging with sparse sampling and iterative reconstruction for ventricular measures: comparison with gold-standard segmented steady-state free precession

**DOI:** 10.1186/1532-429X-17-S1-Q43

**Published:** 2015-02-03

**Authors:** Gabriel C Camargo, Leticia R Sabioni, Fernanda Erthal, Ralph Strecker, Michaela Schmidt, Michael O Zenge, Ilan Gottlieb

**Affiliations:** 1 CDPI - Clínica de Diagnóstico por Imagem, Rio de Janeiro, Brazil; 2 Siemens LTDA, São Paulo, Brazil; 3 Healthcare Sector, Siemens AG, Erlangen, Germany

## Background

Segmented cine imaging with a steady-state free precession sequence (CINE-SSFP) is currently the gold standard technique for measuring ventricular volumes and mass. It requires multiple breath-holds to cover the entire ventricles, thus being prone to misalignment of consecutive slices, time consuming and dependent on breath-hold (BH) capability. Real-time cine avoids those limitations, however poor spatial and temporal resolution of conventional sequences have prevented its routine application. We sought to examine if a newly developed real-time sequence featuring sparse sampling and iterative reconstruction (CINE-RT), which is an investigational prototype, would yield similar results when compared with conventional CINE-SSFP in a group of healthy volunteers.

## Methods

Stacks of short-axis cines were acquired covering both ventricles in a 1.5T system (MAGNETOM Aera, Siemens AG, Germany), using gold standard CINE-SSFP and CINE-RT. Acquisition parameters for CINE-SSFP were: voxel size 1.6x1.6x7.0mm, GRAPPA acceleration factor of 2, temporal resolution of 39 ms, retrospective gating, with an average of 8 heart beats per slice and 2 slices/BH. For CINE-RT: voxel size 1.6x1.6x7.0mm, sparse sampling net acceleration factor of 11.5, temporal resolution of 41 ms, prospective gating, real-time acquisition of 2 heart-beats/slice and all slices in one BH. Left and right ventricle contours were blindly drawn by an experienced observer at end diastole and systole to derive volumes and LV mass.

## Results

Eight healthy volunteers (4 male; 35.2 ± 4.5 years) and twenty two patients (11 male; 44.5 ± 20.1 years) were examined in the same day. All subjects were in sinus rhythm and all images were considered to have diagnostic quality (figure). CINE-RT derived volumes and mass correlated with gold standard CINE-SSFP, with small biases. Table 1 summarizes all results and comparisons.

**Figure 1:**
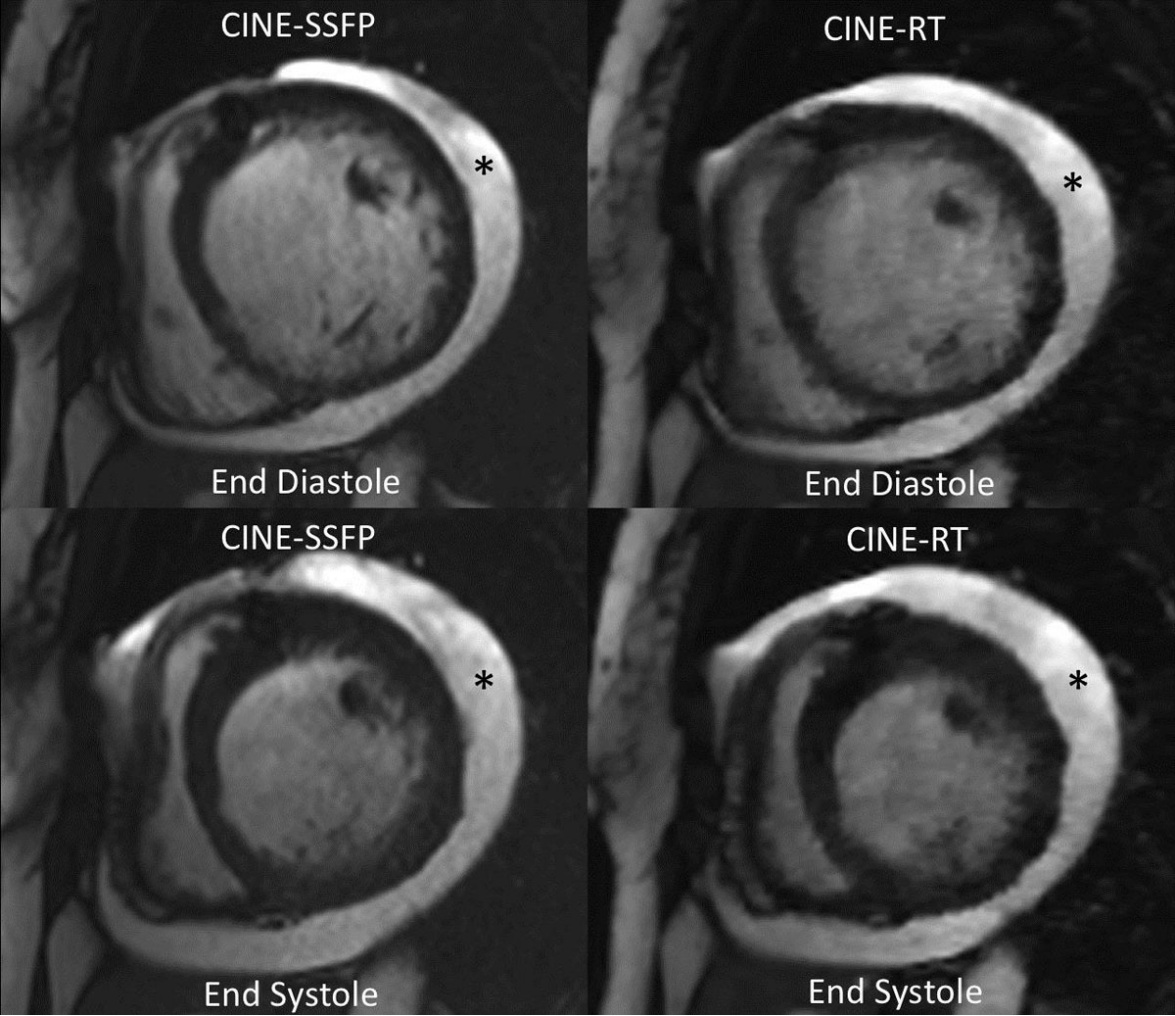
Patient with dilated cardiomyopathy and pericardial effusion (*) seen with CINE-SSFP and CINE-RT.

**Table 1:** 

	LV EDV			LV ESV			LV Mass			RV EDV			RV ESV		
	
	ml ± SD	r	bias ± SD	ml ± SD	r	bias ± SD	g ± SD	r	bias ± SD	ml ± SD	r	bias ± SD	ml ± SD	r	bias ± SD
CINE-SSFP	80.7 ± 24.6	-	-	33.9 ± 20.6	-	-	57.8 ± 15.4	-	-	65.3 ± 12.6	-	-	31.5 ± 7.8	-	-

CINE-RT	73.3 ± 21.8	0.95	7.5 ± 7.6	31.1 ± 19.9	0.97	2.8 ± 4.6	53.4 ± 11.9	0.90	4.4 ± 9.6	58.9 ± 11.6	0.90	6.4 ± 5.6	29.4 ± 8.4	0.82	2.1 ± 4.9

LV: left ventricle, RV: right ventricle, EDV: end-diastolic volume, ESV: end-systolic volume, SD: standard deviation, r: Pearson's correlation coefficient (vs. CINE-SSFP), bias: mean bias (vs. CINE-SSFP).

## Conclusions

CINE-RT with sparse sampling and iterative reconstruction with 2 heart beats per slice achieved spatial and temporal resolutions equivalent to CINE-SSFP, yielding correlated measures of ventricular volumes and mass.

## Funding

Internal.

